# Enhancing Pharmaceutical Packaging through a Technology Ecosystem to Facilitate the Reuse of Medicines and Reduce Medicinal Waste

**DOI:** 10.3390/pharmacy8020058

**Published:** 2020-03-31

**Authors:** Terence K. L. Hui, Bilal Mohammed, Parastou Donyai, Rachel McCrindle, R. Simon Sherratt

**Affiliations:** 1 Department of Biomedical Engineering, School of Biological Sciences, University of Reading, Berkshire RG6 6AY, UK; t.hui@reading.ac.uk (T.K.L.H.); r.j.mccrindle@reading.ac.uk (R.M.); 2 School of Pharmacy, University of Reading, Berkshire RG6 6AP, UK; b.mohammed@student.reading.ac.uk (B.M.); p.donyai@reading.ac.uk (P.D.)

**Keywords:** reuse of medicines, reduce medicinal waste, intelligent pharmaceutical packaging, medicine re-dispensing, theory of planned behavior

## Abstract

**Background**: The idea of reusing dispensed medicines is appealing to the general public provided its benefits are illustrated, its risks minimized, and the logistics resolved. For example, medicine reuse could help reduce medicinal waste, protect the environment and improve public health. However, the associated technologies and legislation facilitating medicine reuse are generally not available. The availability of suitable technologies could arguably help shape stakeholders’ beliefs and in turn, uptake of a future medicine reuse scheme by tackling the risks and facilitating the practicalities. A literature survey is undertaken to lay down the groundwork for implementing technologies on and around pharmaceutical packaging in order to meet stakeholders’ previously expressed misgivings about medicine reuse (’stakeholder requirements’), and propose a novel ecosystem for, in effect, reusing returned medicines. **Methods**: A structured literature search examining the application of existing technologies on pharmaceutical packaging to enable medicine reuse was conducted and presented as a narrative review. **Results**: Reviewed technologies are classified according to different stakeholders’ requirements, and a novel ecosystem from a technology perspective is suggested as a solution to reusing medicines. **Conclusion**: Active sensing technologies applying to pharmaceutical packaging using printed electronics enlist medicines to be part of the Internet of Things network. Validating the quality and safety of returned medicines through this network seems to be the most effective way for reusing medicines and the correct application of technologies may be the key enabler.

## 1. Introduction

Medicinal waste has not only been a problem in the NHS (National Health Service) [1], but also a challenge in other countries in terms of public health, the environment and governmental expenditures [2,3,4]. Trueman et al. [5] reported that £300M of prescribed medicines are wasted every year mainly through medication non-adherence. Together with those unused, unwanted and unexpired medicines, they are major sources of preventable medicinal waste that can currently only be disposed of through managed (e.g., disposal centers at community pharmacies) and unmanaged methods (e.g., domestic sewage, public bins, etc.). One of the ways to tackle medicinal waste is to explore the idea of medicine reuse, which is currently not permitted in the UK [6,7]. A legally approved re-dispensing of medicines scheme has started to work in some areas of the world such as the *SIRUM (Supporting Initiatives to Redistribute Unused Medicine* (https://www.sirum.org/)) originating from the California [8], the *Pharmaceutical donation and reuse programs* operating now in many states of the US [9], and the *GivMed* (https://givmed.org/en/) programme facilitating access to leftover medicines using a smartphone app in Greece [7]. However, there are restrictions to the types and the sources of medicines to be reused since the quality and safety of the returned medicines are not guaranteed [10]. Donating medicines to remote areas that lack resources is another way of reducing medicinal waste through recycling medicines. Nevertheless, the reusing of dispensed medicines is generally not allowed because a proper way of validating the quality of returned medicines is not yet available. Thus, prescribed medicines from individuals are usually not allowed to be donated abroad either [11,12]. A sustainable pharmaceutical supply chain (PSC) management may provide an alternative solution to reducing medicinal waste through the concept of reverse flows. Viegas et al. [13] classifies reverse flows into donation, Reverse Logistics (RL) and Circular Economy (CE), where CE illustrates a close loop supply chain paving the way to reuse returned medicines. The complicated communication flows between a large number of PSC stakeholders could be an obstacle blocking a smooth reverse flow implementation. Pharma 4.0, an extension of Industry 4.0 to pharmaceutical manufacturing, may help establish seamless connections between stakeholders through Internet of Things (IoT) technologies [14,15]; however, the big concern in managing and monitoring the quality of returned medicines still needs to be resolved.

The reuse of medicines is a behavior that can be studied using behavioral sciences [16,17]. Within this perspective, technologies are essential to facilitate attitude change by validating that the medicines returned back to pharmacies have maintained their quality and are safe to use [18,19]. The reuse of prescribed medicines, especially in the UK, is an underexplored research area and the corresponding technologies facilitating this action seem to be an uncharted territory. A structured literature review is reported in this paper to categorize the required technologies applicable to the design of pharmaceutical packaging facilitating the reuse of medicines and the reduction of medicinal waste. Pharmaceutical packaging provides much useful information about a medicine and its use. Additional data regarding its quality and safety which are critical for re-dispensing returned medicines can also be monitored when appropriate technology is applied [20].

Pharmaceutical packaging is regarded as the “key facilitator” for establishing a friendly patient-medication relationship through a patient-centered strategy [21], thus, embedding suitable technologies onto the packaging itself seems to be the best approach for developing the concept of medicine reuse. Manufacturers have already begun implementing technologies into pharmaceutical packaging in order to provide clear information to patients, to protect medicines from the environment, and to cope with changing government regulations and policies [22,23,24,25]. The main targets for applying embedded technologies to the packaging are normally focusing on supply chain management [21,26], anti-counterfeit enforcement [27,28], and quality and safety indications [29,30]. Static technologies dominate previous research on pharmaceutical packaging where the interaction with the package requires an external system such as a RFID (radio frequency identification) reader or barcode scanner using a one-way data transmission protocol. Some of these static technologies may require human interaction to identify their readings such as the TTI (time–temperature indicators) sensing devices extensively used in the food packaging industry [31]. Alternatively, active technologies provide a better package-to-human interaction based on the packaging itself. However, a higher degree of integration of latest digital technologies with the pharmaceutical packaging is required for communicating with the surrounding or remote computing devices. Connection to the Internet using the IoT concept is a new technological trend for telehealthcare empowering a ubiquitous communication with technology embedded pharmaceutical packaging based on cyber-physical systems (CPS) [15,32]. Intelligent packaging, a term extensively used in food packaging, has been implementing both passive and active technologies to inform consumers of the condition of the packaged food [33]. Many technologies used in intelligent food packaging, especially those related to sensing and reporting, can also be applied to pharmaceutical packaging. Emerging multidisciplinary research has enabled technologies to be more effectively applied to reduce medicinal waste through enhancing medication adherence, particularly those studies based on the analysis of human behaviors through a combination of psychology, medication and pharmacy [34,35]. Similarly, it could be argued that the application of technology could influence people to engage in medication reuse by addressing the relevant determinants of intentions to take part in such a scheme in the future. Qualitative studies, as well as the application of the theory of planned behavior (TPB) have previously analyzed intentions and actions towards the returning and re-dispensing of medicines [16,17,18,19], and there are technologies that can help shape user behaviors towards the goal of medicines reuse.

As a precursor to defining a medicine reuse ecosystem, this research conducts a structured literature survey and summarizes the technologies that can be applied to facilitating behavioral changes towards reusing returned medicines. The terms reuse, re-dispense and recycle of medicines are used interchangeably in the current article, distinguishing them from unwanted medicines that need to be disposed of or incinerated, and which will be treated via medicine disposal through waste management. Section 2 describes the structured literature review method used in the searching and screening of peer review papers from popular academic search engines, and how the definitions of inclusion and exclusion are made. The results are presented in Section 3 where a taxonomy of technologies are classified according to the different factors affecting human behaviors. Discussions are made in Section 4 with regard to how the technologies identified in this study can be used to facilitate reuse with their pros and cons further elaborated. A medicine reuse management ecosystem based on the result of the literature review is proposed from a technology perspective and Section 5 explains its structure. Finally, Section 6 concludes the present study and lays down future research directions.

## 2. Methods

A structured literature review was conducted to identify and categorize the available technologies that can help design pharmaceutical packaging to facilitate the reuse of returned prescribed medicines. A rapid scoping review approach based on the PRISMA (Preferred Reporting Items for Systematic reviews and Meta-Analyses) protocol was chosen for the literature survey using a single reviewer, but with awareness of the limitations of not conducting a full multiple-reviewer systematic review [36,37]. The current study focuses on examining a novel concept of implementing appropriate technologies to facilitate the shaping of human behaviors for medicine reuse. PRISMA protocol provided a structured, reproducible and transparent methodology to conduct the article search, and using a single reviewer enabled a rapid review approach which fit the purpose for laying down the groundwork for a future full systematic review of specific studies identified in the present research [38].

Understanding human behaviors is essential in providing healthcare to the general public. Continuous education and constant enhancement of services are essential to influence individual decisions towards planned directions [39]. Previous studies have shown that patients and stakeholders in the pharmaceutical sector generally accept the concept of reusing dispensed medicines as long as certain criteria are met. Bekker et al. [17] investigated patients’ willingness to use recycled medicines, McRae et al. [18] looked at the same issue through the healthcare professionals’ perspective, and Bekker et al. [16] went further to collect the views from all related stakeholders. A more systematic analysis of human behaviors for reuse of medicines in the UK was reported by Alhamad et al. [19], and the three beliefs based on the TPB were studied using a thematic analysis of the associated attitudes after interviewing the local community. The criteria from these empirical studies are similar and the technological requirements are summarized in Table 1.

Pharmaceutical packaging is not the only place for implementing technologies to facilitate the shaping of human behaviors towards reusing returned medicines, associated technologies working cohesively with the sensor embedded packaging are also essential in supporting related activities. Therefore, the literature review for the present study has focused on both the technologies implementable on the packaging and those that extend the embedded pharmaceutical packaging to the outside world such as the Internet in order to share the information with every stakeholder. Table 1 provides the requirements for shaping the stakeholders’ behaviors for medicine reuse based on the qualitative research described previously, and Table 2 illustrates a consolidated version removing duplicates and converting the requirements into keywords for conducting the literature search.

The scope of the current study is limited to the technologies applicable to meeting the quality and safety requirements which are common to all involved stakeholders. However, a brief discussion on how other requirements are tackled can be found in Section 4. Searching of technologies relies on the keywords derived from the requirements through a selection of popular search engines which provide comprehensive listings of journal articles from engineering, pharmacy, medical and psychological sciences. As the purpose of this survey is to lay down the groundwork for deeper systematic review of individual technologies that are appropriate for medicine reuse, the searching formulas were restricted to the titles of papers enabling a preliminary study of latest technologies on recycling medicines. Synonyms for keywords were used to expand the search to a wider area of study; however, the term “pharmaceutical” is not used in some formulas due to the fact that technological research on pharmaceutical packaging is not yet a major research topic for certain technologies. A zero result was obtained in many rounds of keyword searches when the term “pharmaceutical packaging” was in place, so the term was finally removed in some of the search formulas. The five chosen search engines for finding the literature in the present study are: Google scholar (https://scholar.google.com/), Scopus (https://www.scopus.com/), IEEE Xplorer digital library (https://ieeexplore.ieee.org/Xplore/home.jsp), Web of Science (https://wok.mimas.ac.uk/), and Pubmed (https://www.ncbi.nlm.nih.gov/pubmed/).

**Figure 1:**
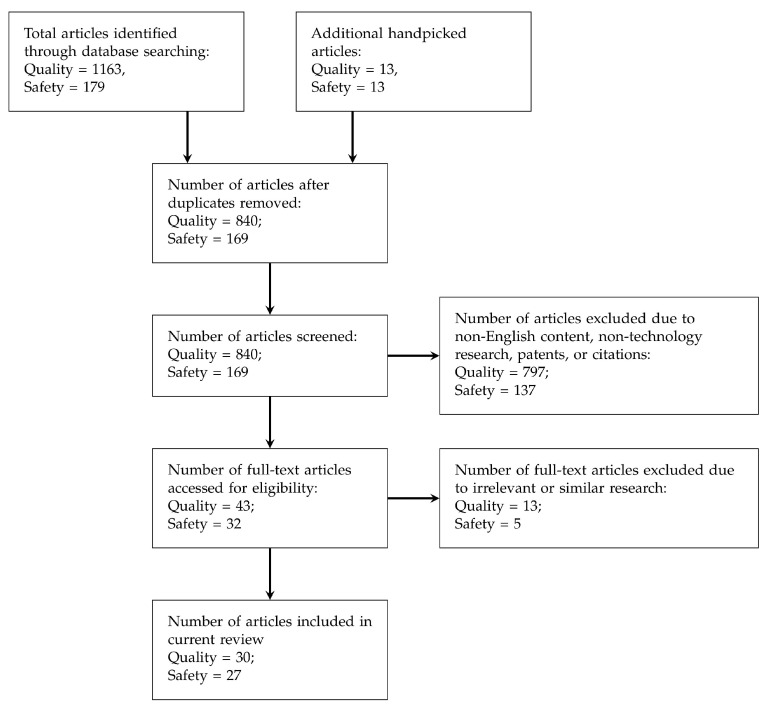
PRISMA [36] flow for screening literature (articles collected are classified into Quality and Safety requirements).

PRISMA flow was followed for screening and selecting the articles to be further studied in this paper, and Figure 1 depicts the selection process flow. The numbers of chosen articles for each process are also illustrated in the flow chart. Other than those academic papers retrieved from the search engines mentioned above, handpicked articles were also collected mainly based on the citations from the collected papers.

## 3. Results

The results of literature review show that the technologies, especially those embedded in pharmaceutical packaging, for reusing medicines returned from patients are still largely ignored by mainstream academic research. Legal issues could be one reason, but the lack of technologies to enable a comprehensive validation of the quality and safety of returned medicines may also be a big obstacle. Law makers, as well as other stakeholders in society, may be skeptical about re-dispensing returned medicines without proper validation [16]. This section describes how latest technologies collected from the literature review can enable the reuse of returned medicines according to the two groups of stakeholder requirements for quality and safety listed in Table 2.

Intelligent packaging has been a major research topic in the food industry and many of its technologies can also be applied in pharmaceutical packaging. The literature review suggests that the main purpose for intelligent food packaging focuses on monitoring the freshness of the food content rather than observing the storage condition of the medicines in pharmaceutical packaging [40]. Deterioration of the packaged food is basically the major concern in the food industry. Müller and Schmid [33] proposes that (i) environmental conditions, (ii) quality characteristics or quality indicator compounds, and (iii) data carriers are the three major concepts in intelligent food packaging. Application of technologies to these concepts, especially the environmental condition monitoring, is closely resembled to the pharmaceutical counterpart where the sensors are measuring the surroundings of pharmaceutical packaging rather than the space inside food packaging. Sensing technologies based on chemical, biological or physical sensors are the core components in intelligent food packaging enabling passive or active indications of the status of the packaged food to consumers [40,41]. Collection of articles was first focused on technologies applying directly to pharmaceutical packaging, but those that applied to food packaging were also chosen in this study when no relevant article was found in the pharmaceutical sector.

Before achieving economies of scale, the high cost of implementation in intelligent pharmaceutical packaging could restrict the application to high priced medicines. However, recycling of the packaging materials has become a trend in protecting the environment and reducing the overall costs in adding technological ingredients into smart packaging [42], thus, the integration of relatively high cost components can be justified.

### 3.1. Technologies for Quality Requirements

Sensors play a crucial part in pharmaceutical packaging for quality assurance of dispensed medicines. The requirements in Table 1 suggest the major quality indicators which detect and report the real-time status of the medicines. Indications include the storage environment (e.g., temperature, humidity, lighting), the handling methods (e.g., contamination, agitation, motion), and the expiration date.

Time–temperature indicators (TTI) are the most popular attachment to an intelligent package reporting the history of the temperature variation for a certain period of time [43]. Specific technologies contribute to the different implementation of the TTI sensing devices with various time scales and sensing technologies for detecting temperature of the storage environment [44] as well as the contents [45]. However, the physical indication of the TTI devices normally requires human intervention through visual inspection. Computer vision based on computational intelligence can replace the human judgment for TTI result recognition but a complicated setup is needed. Mijanur Rahman et al. [46] enhanced the TTI concept using biosensors enabling the detection of the sensing results through a digital interface.

Thin-film technologies through printed electronics or nanotechnology further improve the integration of the pharmaceutical packaging with information technology (IT). Quality assurance indication for the real-time storage conditions can then be shared with all connected stakeholders. Printed electronics allow key sensors such as temperature, humidity and ambient light to be printed on paper or plastic foil. Together with a printed RFID tag, an IT connected storage sensing structure can be built on pharmaceutical packaging [47,48,49,50]. Processing power for performing complicated logical operations is possible by combining prebuilt electronic modules (e.g., microprocessor, other sensors not available yet in printed electronics, etc.) with printed circuits through the hybrid printed electronics methodology [51,52]. Nanotechnology strengthens the thin-film technologies through depositing carbon nanotubes onto the packaging materials and further enhances the manufacturing time and increases the functionality of the embedded electronics for quality monitoring of medicines [53,54,55].

Contamination detection of the medicines inside the packaging is not trivial. Johnston et al. [56] suggested the usage of PT/GC/MS (Purge and Trap/Gas Chromatography/Mass Spectrometry) methods for detecting smoke contaminated packages, while Mielniczuk and Pogorzelska [57] used GC/MS to examine the microbial contaminants. Both proved to be effective, but they are not field-applicable since portable PT/GC/MS machines are not yet generally available. Computer vision could be an alternative for visual inspection of microbial contamination, perhaps under ultraviolet light. However, the resolution for handheld cameras such as those in smartphones may need to be upgraded allowing the general public to conveniently capture and analyze the small particle size of contaminants [58]. An indirect method suggested for identifying potential contamination was to look for visible damage on the packaging [59,60]. Thus, tamper-proof packaging can act as indirect protection from contamination.

Agitation and vibration of the pharmaceutical packaging may affect some medicines, such as insulin [61]. Monitoring of unexpected motions during transportation and storage is therefore necessary to validate the quality for specific types of medicines [62]. The literature search suggests that motion sensing for agitation or spinning applying particularly to pharmaceutical packaging is not being used. No article was found according to the formulas defined in Section 2. However, wearable motion sensors are an emerging topic undergoing extensive research in the personal healthcare sector. Many of them measuring human activities according to variations of the different axis of acceleration or direction can be applied to pharmaceutical packaging as long as they can be flexibly and unnoticeably attached to the packaging materials using thin-film technologies [63,64].

Artificial intelligence combined with image processing enables recognition of the expiry date. Gong et al. [65] illustrated the detection of expiration date on the packaging through a deep neural network, and Peng et al. [66] applied an enhanced “efficient subwindow search” algorithm to locate and recognize the expiry date details from an image of the packaging. QR (quick response) codes combined with SMS (short message service) can be an alternative but a smartphone is required and a predefined standard for QR codes becomes necessary [67]. A dynamic display on the pharmaceutical packaging showing all details of the medicines will be a better way to show all updated information to the patients, and an e-ink (electronic ink) display will be a good low-power (zero power when the display content is stable) method acting as a real-time visual indicator on the pharmaceutical packaging [68]. The flexible e-ink display not only shows the updated information of the medicine inside the packaging, a microprocessor driving the display can also report a real-time quality status according to the sensing results. An electrochromic (EC) display further improves the relatively high-power refresh cycles in e-ink technology during screen content update, and provides a promising alternative for printing a low-power thin-film dynamic display on paper [69,70].

### 3.2. Technologies for Safety Requirements

Safety of medicines is the next critical concern in the reuse process. Even if the returned medicines are quality assured through the technologies mentioned in the previous section, two safety requirements from the stakeholders must be met before medicines could be re-dispensed: tamper-proofing and anti-counterfeiting (see Table 1 for details). Tamper-evident technologies provide indications of whether medicines have been used or adulterated, and counterfeit protection technologies supply methods for authentication.

Tamper-evident pharmaceutical packaging is a mature concept now after the Tylenol tragedy in 1982 where seven patients died due to the intentional adulteration of the medicine [71]. Government regulations enforced the pharmaceutical industry after the incident to implement appropriate tamper-evident and tamper-resistant technologies particularly on pharmaceutical packaging protecting medicines from adulteration [72]. Since then, popular tamper-evident technologies have given indications on broken sealing of the packaging through (i) film wrapping; (ii) blister or strip packs; (iii) sealed pouches and sachets; (iv) tape seals; (v) bubble packs; (vi) heat shrink bands or wrappers; (vii) container mouth seals; (viii) breakable caps; (ix) tear-away caps; (x) sealed metal tubes; and (xi) laminated tubes [73]. Tamper-evident applies also to the external packaging during transportation using RFID embedded film wrap which prevents tampering for the whole pallet of medicines [74]. Tamper-proof packaging must be strong enough to prevent accidental breaking; however, also easy enough to use [75]. To enhance the manufacturability of tamper-proof pharmaceutical packaging in the factories, blow fill seal [23] and IML (in-mold lamination) [76] provide better ways to integrate the tamper-evident sealing into the medicine production process in a single flow but the cost may be higher.

Electronic interfaces allow tamper-proof technologies to be extended to the digital world for automatic recognition of intentional and unintentional tampering. Digital electronics interacting with tamper-evident technologies are still at an early stage, and research examples can be found in relation to blister packs which are the most popular pharmaceutical packaging for tablets by attaching an aluminum film on top of a thermoformed plastic tray [77]. Floerkemeier and Siegemund [78] illustrated the addition of a conductive wire matrix on top of the blister pack where the wires were broken when an individual medicine was removed. The broken wires then activated the built-in communication module to send a message to the patient’s smartphone or a web server registering the usage status of the medicines. This technology is applied to track medication adherence but it can also be used in tamper-proofing. A more advanced tamper-proof solution was demonstrated by Gao et al. [79] who used a controlled delamination material (CDM) as a sealing layer covering the medicines. This CDM layer can be delaminated through activation by electrical power controlled by an RFID tag.

Tamper-proof technologies prevent the pharmaceutical packaging from malicious physical attacks, and also provide indications for potential contamination of the medicines. However, a tamper-evident sealing mechanism will not protect patients from falsified medicines whereas anti-counterfeit technologies can help fight against counterfeiting. Anti-counterfeiting relies on sharing information between suppliers, customers and governments where unique, traceable and unmodifiable identity of individual medicines must be shared on a single platform [80]. Overt technologies, such as holograms and color-shifting paints, usually apply to packaging surfaces allowing trained examiners or even consumers to do visual anti-counterfeiting authentication. These technologies, however, are easily replicated and normally do not last for long. Alternatively, covert technologies such as security taggants and micro-imaging, are basically invisible to naked eyes and require additional tools for examination. Therefore, authentication by normal consumers on covert anti-counterfeiting technologies are restricted. A combination of overt and covert methodologies have been adopted in pharmaceutical packaging to enhance the counterfeit protection strategy from outside of the packaging down to the surface of the medicine, or even inside the individual medicine [81,82].

Anti-counterfeiting technologies can be applied to the packaging materials. Different types of spectroscopy methods, such as Fourier Transform Infrared (FT-IR) or Near Infrared (NIR) spectroscopy can be used to examine the texture of the packaging materials to authenticate the medicine identity [83]. The use of mathematical modeling using discrete Fourier transforms is also possible to perform the authentication by analyzing the texture of the packaging material through an image [84]. Simske et al. [85] proposed a fully variable data printing method applying inks with different visibility under various light spectrums to reject counterfeit medicines.

Tagging technology applicable to anti-counterfeiting has evolved by adding micro-scale taggants directly onto medicines, especially those in the form of tablets or capsules. Printings on the irregular surfaces of the tablets combined with the random minor alignment differences create fingerprints for an individual tag associated with each tablet. A database of these fingerprints can be used as an authentication tool [86]. A biodegradable micro-scale QR code label was proposed by Fei and Liu [87] where the label was attached to the tablet with the code being readable by a smartphone. The QR code can also be debossed on the tablet’s surface through a laser but the depth and the surface materials may affect the reading sensitivity [88]. A microtaggant technology further enhances tagging techniques by using micro-meter scale polymer microbeads with QR tags for on-dose authentication [89]. Reading of the tags may be a destructive process if the reader needs to examine the code on individual tablets, thus, a better reading method should be used for non-destructive examination. Raman spectroscopy provides a non-invasive alternative allowing the recognition of the tags even from the outside of the pharmaceutical packaging [90,91,92].

A proper track and trace system of the medicines from manufacturers to the patients, or multiple patients in case of medicine reuse, is a better way to protect from counterfeiting. A call-in numeric token printed on the packaging can be used to register the medicine once it is used the first time [93], but this method may not help authenticate a reused medicine. Al-Bahri et al. [94] proposed a complete track and trace system based on a central server on the Internet allowing each medicine to be treated as a digital object with unique identity. This DOA (digital object architecture) realizes a shared platform for all stakeholders to retrieve dedicated information when enough cybersecurity is properly implemented. The open and distributed ledger process of blockchain technology enables tracking of medicines registering every transaction among manufacturers, suppliers, pharmacists and patients. The open ledger blockchain can also register the multiple recycling actions between patients [95,96,97].

The Falsified Medicines Directives (FMD) [98] operating in Europe since February 2019 may force the implementation of anti-counterfeiting on pharmaceutical packaging through the addition of QR codes and tamper-proof sealing. However, the certification system may need to be adjusted to fit for a re-dispensing process for medicines reuse.

## 4. Discussion

Technologies for tackling quality and safety requirements can be found from contemporary research but most of them are passive in nature where interaction of medicines with the digital world is missing. The literature review in Section 3 is summarized in Table 3 illustrating a taxonomy of technologies classified according to individual applications and stakeholders’ requirements. Sharing real-time information about medicines between stakeholders is important to maintain a complete medicine reuse system. Storage conditions can be digitally sensed, reported and analyzed dynamically through embedded microprocessors or via cloud computing services. A decision for returning and re-dispensing can be displayed directly on the packaging or indirectly through the smartphone or any surrounding smart devices. A judgment on re-dispensing returned medicines relies on a safety authentication process where the validation of unopened, undamaged and genuine medicines can be performed at pharmacies using dedicated analyzers. Active technologies together with network connectivity empower smart pharmaceutical packaging for the reuse of returned, unused, and unexpired medicines. IoT provides such a platform for sharing information of the medicines through the Internet for every stakeholder, and the concept of a smart object comprising a pharmaceutical packaging with the medicines inside acts as an IoT edge device with digital sensing and network connection [99]. A cloud computing service enables the exchange of information between the smart devices and the stakeholders through wearables, smartphones or full featured computers [100].

A similar structure to that discussed above can be found in a smart medicine box which is an emerging research topic integrating digital sensors and networking capability so that they can monitor normal medicines put inside the box. Additional technologies can be applied to the surroundings of the smart medicine box as well for facilitating an electronic reminder for medication adherence [101], an in-house track and trace system [102], or an interaction with remote servers for telehealthcare [103,104]. Embedding IoT technologies into pharmaceutical packaging allows normal packages of medicines to become intelligent packaging [105,106,107], thus, the requirements for reusing medicines are met where an extension of the real-time information to cloud computing empowers all stakeholders to share data on a single platform. However, three other critical technologies may need to be further investigated to realize an intelligent pharmaceutical packaging for medicines reuse:

(i) Thin-film technologies
  Printed electronics and nanotechnology mentioned previously provide methods to place electronic circuits on packaging materials. However, these technologies are still not common and complicated circuitry such as wireless modules and high-power microprocessors are still not directly printable onto the packaging surface.
(ii) Energy harvesting
  RFID is normally used to provide power to read a passive tag but a continuous power supply for maintaining the regular sensing and the network connection is required. Technology for printed batteries is still in an early stage [108], energy harvesting techniques such as extracting ambient energy could be an alternative [109], and wireless charging can also be a good candidate supplying continuous power to the embedded electronics from a distance [110]. However, all these technologies are not yet mature enough for immediate implementation onto intelligent pharmaceutical packaging.
(iii) Flexible display
  Flexible displays using e-ink or EC technology show a promising way to use minimum energy to sustain a dynamic changing electronic display mounted on existing flat or curved pharmaceutical packaging. Although no power is required for maintaining e-ink screen contents, the irregular updates still require a significant amount of electrical power to align the color pigments. Electrochromism technology reduces the energy for updating EC displays but a regular refresh process is required to keep the screen content visible. New low cost, low energy and printable technologies for pharmaceutical packaging are required.

Other than the two main groups of requirements discussed in Section 3, there are other concerns from the stakeholders in Table 1 to be resolved before an action for reusing medicines can be taken, and they are summarized as below:

(a) patients’ incentive for returning unwanted medicines,
(b) pharmacists’ incentive for extra workload in re-dispensing medicines,
(c) cost effectiveness monitoring of reusing medicines,
(d) legal issues such as legislation on re-dispensing medicines and professional standards for pharmacists,
(e) social norm for promoting medicine reuse,
(f) on-site and off-site collection and distribution system.

Items (a) to (e) are not directly related to technology. However, technologies may help quantify the data (e.g., immediate cost saving for recycling certain medicines, calculation and distribution of incentives to related stakeholders, etc.) or support information exchange in a social networks on the Internet. Social networking may also gather supporting power to influence government decisions on changing policies. Item (f) may make use of the IoT platform to collect, register, authenticate and re-dispense using a proven track and trace system through the IoT networks.

## 5. ReMINDS Ecosystem

Based on the qualitative research within pharmacy practice and the concept of technology integration for pharmaceutical packaging, a group called ReMINDS (Reuse of Medicines through Informatics, Networks and Sensors) has recently been established in the University of Reading with the aim of promoting the reuse of medicines in the UK. ReMINDS is driven by a multidisciplinary team with members coming from pharmacy, computer science and biomedical engineering.

**Figure 2:**
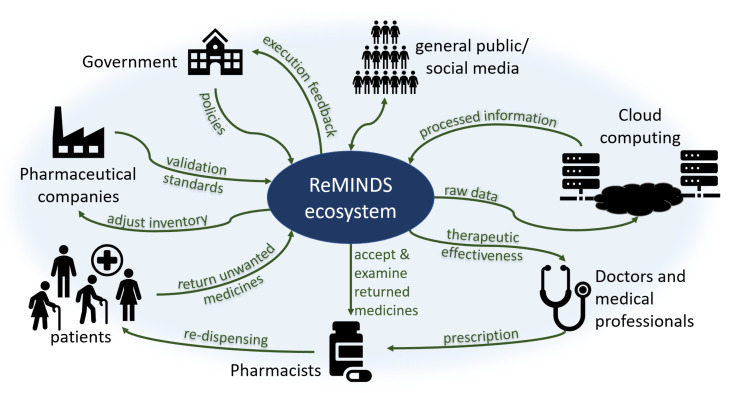
An ecosystem for the reuse of medicines from a technology perspective.

The reuse of medicines is not purely a technical issue since (i) it creates legal concerns involving changes in policies by governments, (ii) it affects commercial decisions involving changes in financial performance for pharmaceutical companies, (iii) it requires voluntary actions involving changes in patient behaviors through patient beliefs, and (iv) it increases extra workloads and risks involving changes in the code of conduct for pharmacists. Previous research suggests that every stakeholder in society contributes part of the responsibility to recycle returned and unused medicines where an ecosystem is apparently established by itself. A novel ReMINDS ecosystem for reusing dispensed medicines through a technology perspective is proposed and Figure 2 depicts the relationship between each party in the hypothesized ecosystem for medicine reuse. The concept of ReMINDS ecosystem can be one of the solutions for reusing dispensed medicines and reducing medicinal waste, and it is built on top of the IoT where seamless connections between medicines and the related stakeholders is the key for success.

Patients and pharmacists are not the only groups in society responsible for taking actions in returning and re-dispensing medicines, other stakeholders in society as a whole play different but crucial roles in maintaining a sustainable ecosystem for reusing medicines. Patients may be the first decision maker to return unused medicines back to the recycle centers, and technologies can provide indications for when and where the medicines are reused or disposed. Pharmacists accept and examine the returned medicines, and technologies enable them to validate the usable conditions before re-dispensing. Raw data of the types, quantity and quality of returned medicines are uploaded to a cloud server empowering an off-site analysis, different entities can retrieve information using various analytical methods. Doctors and healthcare professionals write the prescriptions to the patients but they may not be directly involved in the whole return and re-dispense process; however, technologies allow them to investigate the therapeutic effectiveness based on the information collected and analyzed through cloud computing. Pharmaceutical companies provide standards to pharmacists for validation of the usable conditions for returned medicines, for examples, the duration and limits for out-of-range storage temperature or humidity. Government is a key stakeholder who can set or change the policies enabling and governing related activities, the lawmakers may require specific information from the cloud server to monitor and adjust the execution of policies. As well as playing a role in returning unused medicines, the general public also act as a supporting role through online social networks by influencing the government and establishing a norm for the recycling of medicines.

## 6. Conclusions

A literature survey of latest technologies facilitating the design of intelligent pharmaceutical packaging for reusing medicines is reported. A taxonomy of the reviewed technologies is suggested according to the requirements for shaping human behaviors to take appropriate actions. Through a technology perspective, a novel ReMINDS ecosystem as a suggested solution for reusing returned prescribed medicines based on the literature review is proposed. Active sensing technologies integrated with the IoT platform indicate how a combination of informatics, networks and digital sensors facilitate society to make possible the reuse of medicines.

Technologies provide the tools to directly or indirectly meet the various requirements from each stakeholder. Embedded sensing and reporting electronics on the pharmaceutical packaging help validate the quality and safety of the medicines. Network connectivity helps connect the intelligent packaging globally to all stakeholders in the ReMINDS ecosystem. However, intelligent packaging for reusing medicines is still not mainstream research and more studies in thin-film technologies, energy harvesting, flexible low-power display are essential to empower the technologies on pharmaceutical packaging to become the key enabler for reusing returned prescribed medicines and reducing medicinal waste. Further research on developing and applying appropriate technologies onto and around the pharmaceutical packaging for establishing the hypothesized ReMINDS ecosystem will be one of the aims for the ReMINDS team.

## Figures and Tables

**Table 1:** Technological Requirements for the Reuse of Medicines.

Requirements	Quality	Safety	Others
Patients’ perspective [17]	(1) storage and handling conditions.	(1) tamper-proof packaging; (2) anti-counterfeit.	(1) patient incentive; (2) cost effectiveness.
Healthcare professionals’ perspective [18]	(1) storage conditions (temperature, moisture and light); (2) contamination of package (stain, smell); (3) last dispensing date.	(1) tamper-proof packaging; (2) anti-counterfeit.	(1) cost effectiveness; (2) legal issues regarding pharmacist responsibility, medicine recall, paperwork, efficacy, and governmental regulations.
Stakeholders’ perspective [16]	(1) monitor storage conditions (temperature, light, humidity, agitation, and lapsed expiration date).	(1) anti-counterfeit; (2) track and trace system to the packages for re-dispensed medicines.	(1) patients’ incentive; (2) pharmacists’ incentive; (3) cost benefits shared by stakeholders (patients, pharmacists and health insurance companies).
TPB Behavioral beliefs [19]	(1) storage conditions (temperature, humidity and cleanliness); (2) contaminated packaging.	(1) tamper-proof packaging; (2) errors introduced by patients or pharmacists; (3) anti-counterfeit.	(1) cost effectiveness.
TPB Normative beliefs [19]	Nil	Nil	(1) concern mostly on the social norm for reusing medicines.
TPB Control beliefs [19]	(1) monitor storage conditions (temperature, light, humidity, agitation, and lapsed expiration date).	(1) tamper-proof packaging; (2) anti-counterfeit.	(1) patient incentive; (2) on-site and off-site collection and distribution system.

**Table 2:** Keywords for literature search according to the requirements listed in Table 1.

Requirements	Technologies	Keywords for Search
Quality	(1) storage temperature monitoring (2) storage humidity monitoring (3) storage lighting monitoring (4) storage contamination monitoring (5) agitation monitoring (6) lapsed expiration date monitoring	(1) (intelligent OR smart OR monitor) AND packaging AND temperature (2) (intelligent OR smart OR monitor) AND packaging AND (humidity OR moisture) (3) (light OR optical OR UV) AND food AND packaging (4) packaging AND contamination (5) (vibration OR shock OR acceleration OR shake OR agitation) AND packaging (6) (report OR monitor OR detection) AND expiry
Safety	(1) tamper-proof packaging (2) anti-counterfeit (3) track & trace collecting and dispensing system (4) errors tracking from patients and pharmacists	(1) (evident OR resistant OR detection OR proof) AND tamper AND packaging (2–4) (pharmaceutical OR intelligent OR smart OR packaging) AND counterfeit

**Table 3:** Latest technologies for reusing returned medicines.

Requirements	Technologies
Quality	(i) storage temperature monitoring: passive TTI [43,44,45] (ii) storage temperature monitoring: active TTI with digital interfaces [46] (iii) thin-film technology: printed sensors and RFID tags [47,48,49,50] (iv) thin-film technology: hybrid printed circuits [51,52] (v) thin-film technology: nanotechnology [53,54,55] (vi) contamination detection: PT/GC/MS methodology [56,57] (vii) contamination detection: computer vision [58] (viii) contamination detection: tamper-evident check [59,60] (ix) motion detection: wearable sensors [63,64] (x) expiry date detection: visual inspection [66] (xi) expiry date detection: QR codes and smartphones [67] (xii) on packaging display: e-ink displays [68] and EC displays [69,70]
Safety	(i) tamper-proof: tamper-evident and tamper-resistance on packaging [71,72,73,75] (ii) tamper-proof: tamper-evident for transportation [74] (iii) tamper-proof: implementation during production [23,76] (iv) tamper-proof: built-in digital interfaces [77,78] (v) anti-counterfeit: overt/covert indications [80,81,82] (vi) anti-counterfeit: packaging materials inspection [83,84,85] (vii) anti-counterfeit: tagging on label, on medicine and on-dose [86,87,88,89] (viii) anti-counterfeit: readers for mini-size tags [90,91,92] (ix) anti-counterfeit: track and trace systems through Internet [93,94] (x) anti-counterfeit: open ledger based on blockchain [95,96,97]
